# "Not taken seriously"—A qualitative interview study of postpartum Rwandan women who have experienced pregnancy-related complications

**DOI:** 10.1371/journal.pone.0212001

**Published:** 2019-02-13

**Authors:** Jean Paul Sengoma Semasaka, Gunilla Krantz, Manasse Nzayirambaho, Cyprien Munyanshongore, Kristina Edvardsson, Ingrid Mogren

**Affiliations:** 1 Department of Clinical Sciences, Obstetrics and Gynaecology, Umeå University, Umeå, Sweden; 2 University of Rwanda College of Medicine and Health Sciences School of Public Health, Kigali, Rwanda; 3 Department of Community Medicine and Public Health, Sahlgrenska Academy, University of Gothenburg, Gothenburg, Sweden; 4 Judith Lumley Centre, La Trobe University, Melbourne, Australia; University of North Carolina at Chapel Hill, UNITED STATES

## Abstract

**Background:**

There is limited knowledge on the women’s experiences of pregnancy-related complications in Rwanda. This study aimed to investigate women’s experiences and perceptions of specific complications during pregnancy and delivery and the consequences of these complications on postpartum health and family situation.

**Methods:**

Data were collected through individual in-depth interviews (N = 15). Participants who experienced complications such as postpartum haemorrhage, caesarean section due to prolonged labour/dystocia, pre-eclampsia, or fistula and who were 13–24 months postpartum were invited to participate in the study in July 2015. Interviews were held in Kinyarwanda, digitally recorded, transcribed verbatim, translated into English, and analysed using qualitative content analysis.

**Results:**

Most participants reported that they were previously unaware of the complications they had developed, and they claimed that at discharge they should have been better informed about the potential consequences of these complications. Most participants blamed the health care system as the cause of their problems due to the provision of inadequate care. Participants elaborated different strategies for coping with persistent health problems. Pregnancy-related complications negatively affected participants’ economic situation due to increased health care expenses and lowered income because of impaired working capacity, and participants expressed fear of encountering the same pregnancy-related health problems during future pregnancies.

**Conclusions:**

The findings of this study demonstrate how participants felt that inadequate health care provision during pregnancy, delivery, and the postpartum period was the source of their problems. Participants reported different coping strategies to improve their respective life situation despite persistent health problems. Women’s individual postpartum experiences need to be considered and actions taken at the policy level and also by the local community, in terms of the quality of antenatal and postpartum care services, and in sensitizing the local community about the existence of these complications and preparing the community to support the affected women.

## Background

More than 1.5 million women worldwide are affected by pregnancy and delivery-related complications (PDCs) every year, and the main obstetric complications are postpartum haemorrhage, hypertensive disorders (pre-eclampsia, eclampsia), sepsis/infections, and obstructive/prolonged labour[1]. For every woman who dies of a PDC, at least seven other women will survive such complications [2]. Women who have experienced PDCs constitute a risk group for adverse physical, social, financial, and psychological consequences, and such events might lead to life-long disability [3–5]. In African countries like Benin and South Africa, for example, near-miss cases are between five and ten times more frequent than maternal deaths [6], and in many African countries PDCs affect women’s postpartum life physically, socially, and economically [7, 8]. In addition, for many women the additional obstetric care due to PDCs generates more expenses that can contribute to a cycle of poverty and poor health status. The emergency procedures at the hospital and long hospitalisation periods are often associated with longer times until these women are able to return to work, and such long recovery times can have significant economic impacts [8, 9]. For example, seeking care for PDCs in Benin accounts for about 26% of these women’s average yearly household expenditures. Women are sometimes even obliged to leave the hospital before they have sufficiently recovered because they cannot pay for the health care they still need [6]. In Rwanda, the maternal mortality rate decreased from 1,080 per 100,000 live births in 2000 to 210 per 100,000 live births in 2015, and Rwanda is one of the few African countries that has managed to fulfil the 5th Millennium Development Goal (MDG5) of reducing the maternal mortality rate by 75% between 1990 and 2015 [10, 11].

### Rationale of the study

Little is known about the health outcomes within the first two years postpartum in Rwandan women who have experienced PDCs. The results of this study will assist in filling this knowledge gap and will provide policy-makers and health care professionals with essential information to improve the quality of maternity health services, including the postpartum period.

### Aims

The aims of this study were to determine postpartum Rwandan women’s experiences and perceptions of specific PDCs, including:

- The consequences of PDCs on the women’s postpartum health status and daily life.
- The consequences of PDCs on the women’s family and economic situation.
- The support the women received from health care services and their partners and other family members.

This study is part of the Maternal Health Research Programme (MaTHeR) undertaken by the University of Rwanda in collaboration with the University of Gothenburg and Umeå University in Sweden.

## Methods

### The study setting

The Rwandan health care system is decentralised with the community level as the first, basic level of maternal health services provision [12]. The second level is the health centre, which provides care for pregnant women with uncomplicated pregnancies, whereas complicated cases are referred to either the district hospital level or to the referral hospitals according to the severity of the PDCs [12]. This study was conducted in two locations in Rwanda, the city of Kigali and the Northern Province.

### Subjects and methods

This study used the framework of a previous population-based study in order to identify eligible participants who had experienced PDCs [13]. Purposive sampling was used aiming to explore experiences within the first two years postpartum and perceptions of women who had reported one of four different PDCs: postpartum haemorrhage, prolonged labour/obstructed labour with a subsequent caesarean section, pre-eclampsia, or fistula. A community health worker in charge of maternal health in each village [12, 13] helped to contact the eligible participants and introduced the research team to the participant in the visited village. Eligible participants were individually approached by the first author and orally informed of the purpose of the study, and they signed a consent form prior to the in-depth interview. Fifteen participants were invited to participate in the study, and all of them accepted. Data collection was performed through individual in-depth interviewing. The geographical distribution of participants was as follows: Fifteen participants were residents in either Kigali City or the Northern Province of Rwanda that include in total three and five districts, respectively. The five participants from Kigali City were residents in two of the Kigali City districts and 10 participants were from four districts of the Northern Province of Rwanda. Participants were residents in urban, semi-urban, and rural communities. The closest and farthest village that were visited were 12 km and 112 km from Kigali city, respectively. For the majority of the participants, labour started before they arrived at the neighbouring health centre. As complications were detected, they were transferred from the health centre to the district hospital. Two participants residing in Kigali city delivered in a private hospital.

The time period under investigation included pregnancy, delivery, and the postpartum period until the time of the interview. The interview took place within a time period of 13–24 months after childbirth. An interview guide was composed and included questions on participants’ health status, their experiences during pregnancy, delivery, and the postpartum period, the family and neighbours’ support and attitudes, the support received from health care services, the economic status of the family, and the experiences of motherhood in relation to the pregnancy-related complications.

Prior to the data collection, the interview guide was piloted with two women living outside the study area and revised thereafter. The revision of the interview guide included rephrasing questions using common language expressions instead of medical terms. The individual interviews lasted between 45 and 60 minutes and were held in the participant’s home or at another convenient venue chosen by the participant. Data saturation, i.e. the point where no more significant information was obtained, was reached after approximately 12 interviews, i.e. after three interviews of women with each of the four selected complications. Three more interviews were conducted in order to verify whether saturation had been achieved [14]. All the interviews were conducted by the first author assisted by a female nurse who was an experienced interviewer and no other person could overhear this conversation. The research group had, prior to perform the interviews, contacted the local health authorities and informed about the study in case any referrals would be required. Interviews were held in the local language Kinyarwanda, digitally recorded, transcribed verbatim, and translated into English by an experienced data collector. Field notes were taken during and after each interview, and then used during data analysis and interpretation.

#### Data analysis

A qualitative content analysis, inspired by Graneheim and Lundman [15], was used to analyse and interpret the data. The first author (JPSS), who is fluent in Kinyarwanda, control-read all of the translated interviews while listening simultaneously to the digital recordings and making the necessary language corrections.

The first author (JPSS) is a medical doctor and is familiar with the Rwandan context (i.e. a preunderstanding of the setting). The last author (IM) is an obstetrician with substantial experience in qualitative research and with research experience in the Rwandan setting. As a first step in the analysis, JPSS and IM separately coded one third of the transcribed interviews in English (4/15). Thereafter, JPSS and IM discussed their coding until they obtained consensus [15]. All remaining interviews were inductively coded by JPSS, and some parts were reviewed by IM. JPSS together with IM sorted the codes into content areas based on the codes’ similarities and differences. The codes were categorized into 14 sub-categories and four categories. The analysis process was iterative, moving back and forth between text, codes, sub-categories, and categories [15]. IM surveyed the whole analytic process until its last stage, where categories and sub-categories were shared and discussed between all co-authors until consensus was achieved.

#### Ethical considerations

The research protocol was approved by the College of Medicine and Health Sciences Institutional Review Board of the University of Rwanda (Ref: 010/UR/CMHS/SPH/2014). The WHO guidelines on ethical issues were followed, and participants were informed about their freedom to participate and that they could withdraw from the study at any time they wanted without consequence [16]. Before the interviews, the interviewer (JPSS) provided information about the study to the participants. All participants gave verbal and written consent before their participation in the study.

## Results

Participants were 21–39 years of age (mean age 28.5 years). The majority of participants had completed primary or secondary school, and only two participants reported no education (Table 1). The majority of the participants were married, and two participants were separated. Eight participants reported a monthly income less than 35,000 RWF corresponding to approximately 45 US dollars (Table 1). All participants had had at least two antenatal care visits during pregnancy, and they had all delivered at a health care facility. Table 1 presents the background characteristics of the participants. An overview of the results is presented in Table 2 with an overall theme, four categories, and their 14 sub-categories. The theme “Experiencing challenging health problems necessitating reliance on own resources postpartum” was identified during the analytical process and captured the summarized experiences and perceptions of the participants. The four categories contributing to the theme were titled “Being unknowledgeable and unprepared for pregnancy complications”, “Blaming the health care system”, “Developing coping strategies for a better life”, and “Hope and fear of the future”. Each category is briefly summarized below followed by a presentation of the content of each sub-category, including relevant quotations

**Table 1:** Background characteristics of participants.

No	Time postpartum (months)	Number of ANC* visits	Number of children including index child	Women’s education	Household monthly income	Marital status
**Women who experienced fistula**						
01	20.7	4	6	Completed primary school	<17,500 RWF**	Married
02	23.3	4	1	No education	<17,500 RWF	Widow or separated
03	16.61	2	3	Completed primary school	Between 17,500 and 35,000 RWF	Widow or separated
04	17.73	4	2	No education	Between 17,500 and 35,000 RWF	Married
**Women who experienced pre-eclampsia**						
05	23.95	2	3	Completed secondary school	Between 17,500 and 35,000 RWF	Single
06	14.74	4	5	Completed primary school	<17,500 RWF	Married
07	13.91	4	1	Completed secondary school	Between 17,500 and 35,000 RWF	Single
**Women who experienced caesarean section for prolonged labour**						
08	15.03	4	1	Completed secondary school	>35,000 RWF	Married
09	***	4	1	Completed secondary school	>35,000 RWF	Married
10	21.48	3	3	Completed secondary school	>35,000 RWF	Married
11	21.48	4	1	Completed primary school	>35,000 RWF	Single
**Women who experienced postpartum haemorrhage**						
12	18.98	3	3	Completed primary school	>35,000 RWF	Married
13	16.18	4	2	Completed primary school	Between 17,500 and 35,000 RWF	Married
14	18.09	4	2	Completed secondary school	>35,000 RWF	Married
15	24.14	4	1	Completed primary school	>35,000 RWF	Married

*ANC: Antenatal Care

**RWF: Rwandan Francs

***Missing data

**Table 2:** Theme, categories and their sub-categories.

Theme	Categories	Sub-categories
**Experiencing challenging health problems necessitating reliance on own resources postpartum**	**Being unknowledgeable and unprepared for pregnancy complications**	• Unawareness of pregnancy-related risks and associated health problems • Trying to grasp the health problem • Being abnormal and restricted
	**Blaming the health care system**	• Not taken seriously by health care providers while seeking care • Unavailability of required health care services
	**Developing coping strategies for a better life**	• Trying to remain a good mother • Coping with poor economic situation because of health problems • Developing coping strategies to improve the situation • Enduring criticism from family and community • Support from one’s husband is crucial for enduring the situation • Dealing with challenges in sexual life
	**Hope and fear of the future**	• Complications influencing future pregnancies • Mixed feelings of fear and hope • Putting hope in God

### Being unknowledgeable and unprepared for pregnancy complications

Complications were often unexpected and sudden. Almost all participants had been discharged without being properly informed about the complications that they suffered from. They underwent a process of trying to understand what, how, and why the complications had occurred in order to make sense of the situation. After improved understanding of their adverse health situation, some of the women felt helplessness and experienced a sense of being abnormal.

#### Unawareness of pregnancy-related risks and associated health problems

Most participants were previously fairly unknowledgeable about the complications that they had developed. For some i.e. pre-eclampsia cases, they had reported having a series of symptoms and signs that were not noticed or discovered at a routine antenatal care visit.

*‘I didn’t know much about the sickness*. *I felt confused*, *felt like I was going to die*. *Others started saying that it was poison*. *I didn’t know much about such a sickness*.*’* (Pre-eclampsia; PE)

Other participants experienced having complications without understanding the nature of the problem, and some participants did not even know that such conditions and complications even existed.

*‘I felt desperate and felt like I was the only one with that kind of problem*, *because*, *when I reached the hospital*, *I saw them wondering what it was*. *As if it was a new kind of disease that I had*, *one that they had never seen before*.*’* (Fistula; F)

Additionally, participants had been discharged without being properly informed about the complications they were suffering from and potential problems to expect during the postpartum period.

*‘After performing a caesarean section the medical doctor could see how he can discuss with you and tell you that a woman who has a caesarean section can have this kind of problem*.*’* (Caesarean section; CS)

#### Trying to grasp the health problem

The process of making sense of the situation was necessary in order to understand both the series of events that resulted in the complication and the magnitude of their health problem related to these complications, as well as to understand the prognosis of their health problem (to know if it will heal, worsen or not).

*‘Sometimes I try to stop taking medicines like for one week and see what happens*, *and after that if I feel like it is increasing again; then*, *at that time I take the medication again*.*’* (PE)

Some participants preferred to ask other women who had experienced similar health problems, and then they compared their problems to the other women’s health issues. This was valuable for their understanding of their adverse health situation.

‘*When we meet* [other women with fistula] *we discuss and share experiences*, *and we are free to talk about our cases because we are among ourselves*. *This makes me feel that I am not alone*.’ (F)

Other participants considered their problem as a “normal” problem common to all postpartum women, and they considered that this situation to be permanent, which resulted in that they stopped seeking health care.

*‘I didn’t seek care because I was thinking that it is normal for women who have had caesarean section to have this problem*, *and some women told me that this is the common case for all women with c-section*.*’* (CS)

#### Being abnormal and restricted

Some participants expressed feelings of not being normal women and felt restricted in what they could do in their daily life.

*‘Before this disease*, *I used to visit many people*, *but now I can’t even take a long trip*, *it is impossible*. *I used to go to church*, *but now I can’t*.*’* (F)

The PDC and its consequences had had a profound impact on their postpartum lives. It was also reported that their lives had gradually deteriorated including a feeling of being hopeless.

*‘But for me I am feeling desperate*, *like when I get sick I feel like my death is coming soon*, *and I start to think about these children* [own children] *and start to wonder what will happen if I die because I am the* [only] *one who is looking after them*. *I feel like […] there is no hope for the future*.*’* (PE)

### Blaming the health care system

Participants reported low quality and insufficient access to services during pregnancy, delivery, and the postpartum period in the Rwandan health care system. Some participants complained that being neglected during labour and delivery was the cause of their later complications. Others complained about a lack of attention from health care providers in the postpartum period when they sought care for their health problems. Participants also reported on the lack of needed health services in the postpartum period.

#### Not taken seriously by health care providers while seeking care

Participants thought that the health care system failed to prevent or to discover their health problems at the time.

Most participants considered the health care system to be the cause of their health problems during pregnancy and delivery due to the provision of inadequate health care.

‘*I was neglected when I was giving birth*. *When I entered the maternity ward*, *there was no electricity*, *and the ambulance had just left with another pregnant woman*. *There were two nurses that were telling me to stay in the maternity ward* [participant crying]. *They were looking at me and laughing at my clothes*. *When the ambulance came back*, *the who nurse was in the ambulance removed me from the ambulance and said*: *Please bring her back* [to the maternity ward]. *I am going to try to help her to deliver*. *When in the maternity ward*, *she performed an episiotomy and I delivered after that*. *She told them that “you have neglected this mother*, *why didn’t you help her*? *If you had performed an episiotomy a little bit earlier she would not have suffered like this* !!’ (F)

Later, during the postpartum period when the participants returned to a health care facility seeking care, they reported a general lack of attention by health care providers regarding postpartum women's health issues, resulting in not receiving the expected health care services.

*‘I was not satisfied with the health care received*, *but he* [health provider] *told me that*, *if that symptoms continued*, *I should come back and he would see if he could give me same medication to help me*.*’* (CS)

#### Unavailability of required health care services

Participants complained about not having access to the required health care services when they were in need of them. Some participants were not able to pay for the community health insurance, which would have allowed them to access the more advanced health care services that they required and to medical doctors in district hospitals. Therefore, they only had access to nurses at health centres who sometimes were not sufficiently skilled to provide the needed help. Some participants were not aware of the existence of health care services designed for specific health problems, for example, fistula problems.

*‘I am very tired of returning to that health centre*. *I want now only to go to the hospital… Because I do not have health insurance and do not have money to pay for it since last year*.*’* (F)

*‘Health care services are expensive for others* [women] *who don’t have the health insurance to be able to go to the advanced-level health facility […] at the health centres there are no medical doctors to help women who experienced complications [PDCs]*.*’* (PE)

### Developing coping strategies for a better life

Most participants had the ability to adapt their lives to their new circumstances. They tried to find ways and strategies to help them improve their situation, including adjusting their own attitudes and behaviours. Coping strategies could also include putting significant efforts into hiding their health problems and trying to appear as normal as possible. In addition, some participants had to face financial difficulties because of health care expenses or because their health condition was not allowing them to work and earn an income.

#### Trying to remain a good mother

Some participants were still suffering from the consequences related to their PDCs. They often felt powerless because their poor health status did not allow them to take care of their children as they wished. Despite this situation, they were trying their best to take care of their children or doing their best to appear as good of a mother as possible in the eyes of their neighbours and other family members.

*‘Sometimes I condemn myself about that*, *but always I try to do my best*, *for example*, *here at home*, *even if it is not easy I try to carry him* [the baby] *on my back*, *but if I have to walk*, *I cannot do it*. *Even if people will criticize me*, *I don’t care about it*. *But for a small walk while trying to make him sleep*, *I can do it*. *But you understand that he really needs the affection*, *and he misses being carried on his mother’s back*.*’* (CS)

#### Coping with poor economic situation because of the health problem

All participants were negatively affected financially by their PDCs and suffering from PDCs often resulted in unexpected and unplanned expenses, sometimes even expenses for burying a dead baby. Some women had spent all of their savings, and were obliged to sell a plot of land, or had to take a loan from the bank or from friends in order to pay the hospital bill and other health-related expenses.

*‘He* [the husband] *requested a loan of one million* [Rwandan francs] *when I was hospitalised*, *and we spent almost 2 months there and when we left*, *we had spent almost all the money*. *Having paid the hospital*, *the burial ceremony for the first child*, *and also for my first follow-up*, *we could not buy anything else*.*’* (PE)

Due to the huge expenses, some participants fell into a cycle of poverty because they were not able to recover sufficiently to start working and earn an income.

*‘They say that this woman* [me] *is already handicapped and she is no longer able to do anything*. *You cannot hire her to do some work for you*. *So*, *for me it is this that has led me to despair*.*’* (Postpartum haemorrhage; PPH)

#### Developing coping strategies to improve the situation

Participants tried to manage their situation and avoid factors that could exacerbate their adverse health conditions, thus adapting their behaviours. For example, participants with fistula developed various strategies of using diapers, butter, powder, and perfume in order to hide their problem and to not let their entourage become aware of their problem.

*‘I try to protect myself using diapers made by old clothes covered with plastic*, […] *I go and buy the plastics baby’s diapers* […] *I clean* [them] *every time and cannot use a dirty tissue before I clean it*. *I wash my tissues every time and I use perfumed soap and also*, *I put powder on it*.*’* (F)

As part of such strategies, some participants tried to predict or anticipate the negative consequences that could occur while taking into account their own capacity and limitations. One strategy could be to hire someone to help them.

*‘Sometimes I tried to do small and easy activities*, *but you understand that it is not as it was before*. *Sometimes I have to stop*, *and now I need to hire someone to help me to do things that I used to do myself before I got this problem*. *When I have high blood pressure*, *I can’t work anymore or do small things like going to the market or to church or visiting friends*.*’* (PE)

Participants also tried to avoid having to walk long distances or carrying things on their head in order not to exacerbate their health problems. Others limited long visits to family members or limited their presence in public in order to avoid anyone discovering their problem.

*‘There are some jobs that I am not able to do*, *but other easy jobs I can manage to do*. *Like*, *for example*, *when I go to cultivate in the fields*, *I immediately have urinary incontinence*, *and this makes me uncomfortable and the urine burns me and does not allow me to continue*. *Even when I carry heavy things I have the same problem*, *and it requires me to go and change my diapers immediately when I arrive where I was going*.*’* (F)

#### Enduring criticism from family and community

Most participants described how being physically affected by their PDC implied limited work capacity and changed behaviour in order to adapt to the complication. Because of these changes in their behaviour, they were often exposed to criticism from family members and neighbours. Participants felt that they were being judged even though others were unaware of their specific health problem, their suffering, and the negative health consequences they experienced. Participants also felt accused of being lazy and “bad mothers” because they did not behave like other “normal women”.

*‘My neighbours told me that I had become very lazy*. *They say that I am spending all my days lying down not doing anything because they are not aware of my problem*. *They make me very sad because they do not know me or know what my conditions are*.*’* (CS)

This situation resulted in some of the women being isolated from the rest of the society, and they often withdrew from the community out of shame. Others isolated themselves out of fear of being stigmatised.

*‘I really live alone and I don’t have any friends*. *I started to prefer to be alone immediately after I got this problem’*. (F)

#### Support from one’s husband is crucial for enduring the situation

The husband’s attitude after a woman had developed a complication varied. Some husbands supported their wives, and this helped the women to remain emotionally stable and to physically handle their health problem.

*‘He* [the husband] *was afraid*, *too*, *but he was telling me to be hopeful based on the judgement of the medical team*, *which was telling us that there is a possibility that I can be treated and healed…Based on our family living standards*, *he did the best he could*. *He didn’t let me become helpless*.*’* (F)

*‘He* [the husband] *supported me very much*. *He was the one who was with me at the hospital*. *He even asked for his annual leave*. *Even when the child died*, *we came back together without other problems*. *He didn’t leave me alone*.*’* (PE)

However, other husbands abandoned their wives or left them with very little support. Abandoned women often lived in extremely poor situations because in addition to their health problems, these women also had the child-caring burden alone.

*‘I have delivered without being with my husband*, *and up to now*, *apart from calling and saying that he will come*, *that is the only thing he is doing*. *If I considered how I have suffered alone*, *I don’t need any husband at all*. *What I am focusing on now is about my future*, *how I can have a better life and how I will take care of my child*.*’* (PPH)

#### Dealing with challenges in sexual life

Some participants had not recovered physically and had also experienced dyspareunia and decreased libido. Most of them reported that they were trying to abstain from intercourse. However, in order to remain a good sex partner, there were some participants who tried to continue to participate in sexual intercourse despite sexual problems in order to satisfy their partner.

*‘I sometimes have sexual intercourse with my husband*, *but most of the time I feel like my body does not want this*.*’* (F)

‘*Sometimes I accept* [sexual intercourse] *and suffer just to avoid making him* [the husband] *sad*. *It is not good to make the same excuse every day*.’ (CS)

### Hoping and fearing the future

Generally, participants were uncertain about their futures and about the outcomes of future pregnancies. However, despite this uncertainty, some participants believed that modern medicine could help them to be healed or they could rely on God’s intervention for their healing.

#### Complications influencing future pregnancies

Some participants reported that before the complication occurred, they had planned to have more children. However, due to the pregnancy-related complications some wanted to prevent or to delay a future pregnancy by using contraception. A number of participants were still suffering from complications and feared having the same experience in a future pregnancy.

‘*I am done*, *and I am going to prevent pregnancy and not give birth to another child*. *From my point of view*, *I do not plan to have more children*.’ (CS)

#### Mixed feelings of fear and hope

Some participants expressed that they had been permanently handicapped by the complication, and they doubted the possibility of regaining future health. At the same time, however, it was common that they had the hope to be cured and to return to a normal life at some time point.

*‘I just feel like waiting for death*, *… What else can I wait for*? *I just worry about this youngest* [child]. *The others* [older children] *are old enough to go work for others and earn their living if I would die*.*’* (PE)

‘*I believe that I will be normal again*. *I hope that one day I will be able to get rid of all the diapers and things I need in order to deal with my problem*.’ (F)

#### Putting hope in God

Despite their problems, some participants still had trust in God. They believed that they had survived because of God’s intervention, and they were still counting on God to heal them and help them to recover. Some participants had used all possible human means to seek care and were still not cured, and they expressed hope that God would come to their aid. Other participants were pessimistic because they were not able to access the required care.

*‘I think that I could have died*. *It is only God who helped me*, *otherwise I could have been worse*.*’* (PPH)

*‘Also*, *the medicines help to reduce the problem*, *and I believe that God will heal me one day*.*’* (PE)

## Discussion

The aim of this study was to investigate women’s experiences and perceptions of specific complications during pregnancy and delivery and the consequences of these complications on postpartum health and family situation.

A main finding in this study was that participants commonly lacked knowledge and were unprepared for the risks and health consequences of complications related to pregnancy and delivery. In addition, the participants were generally not satisfied with the accessibility and quality of the health care they received during pregnancy, delivery, and the postpartum period. However, participants commonly developed different coping strategies, including strategies for hiding their health problems from others. In general, all of the participants had suffered economically from their PDCs due to high health care expenses, debts, and loss of productive capacity.

The participants had attended the maternal health services i.e. made at least two antenatal care visits, and all of them delivered with the assistance of a health professional, either a midwife, a nurse or a medical doctor. However, the participants were complaining about the lack of adequate health care services and the quality of the health care received during pregnancy, delivery, and postpartum. Many of the participants also reported that they had not been taken seriously by health care providers, and some felt seriously neglected. A previous study from Rwanda has demonstrated similar results reporting bad attitudes in the health care provided during labour, such as disrespectful care, verbal and physical abuse, shaming, humiliation and insults [17]. Poor quality of care, unfriendly health care providers, and inadequate health care services have previously been identified as barriers to maternal health care utilization [17–20]. Thus, the experiences of poor quality of maternal services may in the long run become one of the main barriers that would prevent Rwandan women to attend maternal health services. Severe maternal morbidity might be sudden and unexpected [21], and our participants reported being unprepared and lacking information about the risks and consequences of PDCs. In addition, the participants were often discharged from the health care facility without being adequately informed about the complications they had developed. Studies done mainly in developed countries have reported how important it is with debriefing interventions performed at the time of discharge, in order to prevent psychological trauma, to provide practical information, and to support women in the postpartum period [22–24].

Our participants described how they developed different strategies to manage their health problems, both in relation to how to cope with the adverse health consequences and with insufficient support from the health care system but also how to anticipate and avoid the family comments and complaints. The participants felt that they were exposed to criticism from both family members and their neighbours because they were not behaving as expected. The participants often refrained from communicating their health problems or they made efforts to hide their symptoms in order to avoid criticism from others, but these strategies heavily restricted the participants’ lives. Similar findings have been reported in Bangladesh, Democratic Republic of Congo, Ghana, and Uganda, showing how women suffering from PDCs become isolated and are obliged to limit social interactions as much as they can [25–27]. In this study some participants experienced challenges in their sexual life, and some reported dyspareunia or decreased libido. Some participants tried to abstain from intercourse, and others, in order to remain a good sexual partner, tried to continue to participate in sexual intercourse despite physical and sexual problems. The same experiences of challenges in satisfying partners’ sexual needs, sometimes resulting in divorce, have also been identified in other studies with similar settings. Studies done in African countries have shown how women experiencing fistula are often abandoned, abused, and ostracised by their husbands[26, 28–30]. In our study however, some of the participants were fully supported by their husbands. Rumination and self-blame have been identified as a common psychological response among women, following the experience of severe maternal morbidity [31, 32]. Our participants reported going through a process of trying to reach an understanding of what had happened to them and why this complication had occurred. This process helped the participants to make sense of the situation and to move forward. A similar process was also described in a review of qualitative studies and by a study from the UK, where women used the internet after they had returned home in order to search for information to understand what had happened to them [23, 32]. In the review study, it was furthermore reported that experiences of severe maternal morbidity were seen by women as a consequence of their own mistakes they had made during their pregnancy, or as a punishment for their bad behaviour [32]. On the contrary, the majority of our participants blamed the health care system for inadequate health care, while a few attributed their adverse health problems to their “weak body”. Alternatively, they considered their problems as “normal”, and as experienced by all postpartum women. To identify and avoid factors that exacerbate a specific problem is a common strategy that has been presented in other studies examining women who suffered from postpartum haemorrhage or fistula [23, 25]. This study highlights the vulnerability of participants who made great efforts to hide their symptoms and adverse health conditions in order to avoid stigma and criticism from their neighbours and others. These participants felt restricted because their health conditions, including sexual difficulties, and the feeling of shame, did not allow them to fulfil their roles in the household and the community. Almost similar situations have been reported among women experiencing PDCs in Bangladesh, Democratic Republic of Congo, Ghana and Uganda, highlighting how these women might become isolated with a tendency for them to limit social interactions as much as possible [25–27]. Our participants, suffering from fistula-related complications, managed to find means that helped them to hide the urinary leakage. Similar results have been shown in other studies that describe almost the same coping strategies[25, 26].

The participants described difficult experiences like fear of dying and fear of losing a baby, and even losing a baby, and severe postpartum health problems. They also described uncertainty and doubts about their future prospects and feelings of loss of dignity and decreased self-esteem. For some of the participants, their complications had totally changed their life situation, and they felt miserable and had no hope that their situation would improve in the future. Similar results have been reported in other studies [21, 33–35]. The majority of the participants described a hope that God would help them to endure their ordeal and would ultimately heal them. Religion and spirituality have previously been found to be comforting factors for women who have experienced near-miss complications during pregnancy and delivery [21, 23, 33].

Participants who had experienced support from their husbands and other family members described this as a significant factor for successfully managing their postpartum health issues. Emotional support, especially support from the husband or other family members, has been shown to be very important for women in these situations [27, 34, 35]. At the time of the interview, some participants reported still being physically affected by the consequences of their PDCs. Self-rated poor health status has also been reported in previous studies [21, 23, 33]. Such health problems might thus impair the woman’s daily life situation, and moreover the health problems might also negatively influence her economic situation and thus have an impact on her whole family. For some participants, all of the family savings had been spent on the hospital costs and subsequent costs related to the postpartum health issues. These health problems often resulted in decreased working capacity that in turn meant decreased household income. Thus, the participants often felt trapped in a vicious circle of increased expenses for health care along with less productivity resulting in less income, thus making the family poorer. Similar results have been found in other studies demonstrating how women with pregnancy-related complications have lost their productive capacity and incurred financial strain with an apparent risk of falling back into poverty [8, 27].

## Methodological considerations

One strength of this study is that purposive sampling was employed targeting prevalent and important PDCs, and this approach most probably yielded a comprehensive and deep understanding of these phenomena from the perspective of the participants in this study. The fact that the interviewer is a medical doctor and a man might have resulted in a power imbalance during the interviews, but on the other hand, the trust in medical doctors is high in the Rwandan society. In addition, many sensitive issues were disclosed by the participants, therefore, we do not consider this to have been a major problem. An experienced female nurse interviewer was also present during the interviews to ensure that the participants would feel comfortable in the situation. Triangulation increased the credibility of the study. This was undertaken by two investigators performing coding separately (i.e. JPSS and IM), discussing their respective findings (results), and thereafter reaching consensus. Furthermore, other authors in the research team who were not familiar with the setting provided an external perspective during data analysis and interpretation. Transferability of the findings was achieved by the fact that our study was undertaken in the northern province of Rwanda and in Kigali city. Kigali city represents the urban area while the northern province represents the semi-rural and rural areas. Therefore, we believe that our findings most probably reflect general (a large part of) experiences of Rwandan women, suffering from severe complications related to pregnancy and childbirth (who have experienced the selected pregnancy-related complications). Confirmability was ensured by having a research team including researchers with different professional backgrounds, various pre-understanding and experiences, jointly discussing and interpreting the findings until consensus was obtained [36]. In addition, keeping field notes on informal communication increased the dependability of this study.

## Conclusions

This qualitative study described experiences of postpartum Rwandan women who had experienced PDCs. The findings point at serious pregnancy and delivery-related complications that were neglected by the health care services. Women felt abandoned and handicapped, were unable to return to work tasks, and incapable of fully taking care of the family, which also negatively influenced their psychological health and economic situation. The findings in this study underscore the importance of taking individual women’s experiences into consideration, as it fills a knowledge gap and builds up for confirmative epidemiological studies. However, it also calls for actions to be taken at the policy level and by the local community. The Ministry of Health needs to be alerted on the insufficient quality of antenatal care, delivery and postpartum care services, while the local community needs to be aware of that such complications may occur and be prepared to support the affected women.
